# Development of a Knowledge Graph for Automatic Job Hazard Analysis: The Schema

**DOI:** 10.3390/s23083893

**Published:** 2023-04-11

**Authors:** Sonali Pandithawatta, Seungjun Ahn, Raufdeen Rameezdeen, Christopher W. K. Chow, Nima Gorjian, Tae Wan Kim

**Affiliations:** 1 Sustainable Infrastructure and Resource Management, UniSA STEM, University of South Australia, Adelaide, SA 5000, Australia; 2 Department of Civil and Environmental Engineering, Hongik University, Seoul 04066, Republic of Korea; 3 Sustainable Infrastructure, South Australian Water Corporation, Adelaide, SA 5000, Australia; 4 Division of Architecture and Urban Design, Incheon National University, Incheon 22012, Republic of Korea

**Keywords:** knowledge graph, ontology, ontology schema, job hazard analysis, safety management, implicit knowledge, explicit knowledge, METHONTOLOGY, construction safety, knowledge management

## Abstract

In the current practice, an essential element of safety management systems, Job Hazard Analysis (JHA), is performed manually, relying on the safety personnel’s experiential knowledge and observations. This research was conducted to create a new ontology that comprehensively represents the JHA knowledge domain, including the implicit knowledge. Specifically, 115 actual JHA documents and interviews with 18 JHA domain experts were analyzed and used as the source of knowledge for creating a new JHA knowledge base, namely the Job Hazard Analysis Knowledge Graph (JHAKG). To ensure the quality of the developed ontology, a systematic approach to ontology development called METHONTOLOGY was used in this process. The case study performed for validation purposes demonstrates that a JHAKG can operate as a knowledge base that answers queries regarding hazards, external factors, level of risks, and appropriate control measures to mitigate risks. As the JHAKG is a database of knowledge representing a large number of actual JHA cases previously developed and also implicit knowledge that has not been formalized in any explicit forms yet, the quality of JHA documents produced from queries to the database is expectedly higher than the ones produced by an individual safety manager in terms of completeness and comprehensiveness.

## 1. Introduction

The construction industry accounts for approximately 20% of all occupational fatalities [1]. High rates of injuries and fatalities have been a persistent problem in the industry and put construction companies and managers under great pressure for improved work safety in modern construction projects. Such high demand for improved safety, especially in more developed countries, created a strong need for a systematic approach to construction safety management, often referred to as a “Safety Management System (SMS)” [2]. An SMS is identified as a set of multidimensional integrative efforts for safety, including site management planning, hazard identification, risk mitigation, project safety rules and policies, site inspection, training, consultation, worker engagement, accident investigation and analysis, and safety performance evaluation [2,3,4]. As such, an SMS is more than just a ‘‘paper system’’ of policies and procedures [5], and it is viewed as a mechanism integrating such multi-pronged efforts for safety into the project organization’s management goals and daily processes at the site [6,7].

Job Hazard Analysis (JHA), also known as Job Safety Analysis (JSA) [8] or Safe Work Method Statement (SWMS) [9], depending on the region, is regarded as one of the essential elements of an SMS [10]. JHA is a proactive approach to identifying, evaluating, and controlling safety risks, and it involves dividing an activity into detailed tasks, identifying all hazards associated with each task, assessing the risks, and deciding on the most appropriate risk reduction technique for high-risk factors [11]. As such, JHA provides a methodology to bring reality into proactive discussions about how to prevent accidents at the workplace before and during work [8]. Researchers found that JHA also brings about improvements in procedures, methods, protocols, quality of materials, equipment, tools, the standard of training of employees, and the identification of environmental problems and issues, not only a reduction in the number of accidents [10].

However, the proper preparation and application of JHA require significant staff time, effort, and expert knowledge about various construction tasks and their risks. Additionally, since each construction project has unique characteristics in terms of work environment, construction methods, safety budget, the experience of construction workers, etc., it is quite challenging to conduct a thorough JHA with all the site-specific, temporary conditions considered [12]. Furthermore, construction workplaces have a dynamic nature, including changes in design, changes in construction methods, and changes in the work environment, making it even more challenging to reflect all those transient factors in JHAs [13]. Additionally, while compliance is of the utmost importance in any construction project, it is challenging to ensure that the safety measures proposed in a JHA are all compliant with the relevant codes and regulations due to the complexity of individual situations at a site [8,14].

In the current practice, the development of a JHA is a manual process and thereby highly influenced by the experience and knowledge of the safety personnel who develop it [15]. Due to such a reliance on individual staff members’ knowledge and experience about the construction work and their ability to predict site conditions, identify risk items, and propose appropriate risk reduction techniques, a JHA can easily be incomplete, incorrect, or biased [16,17,18]. With these challenges and difficulties in producing appropriately developed JHAs, a number of companies in the construction sector were found not fully compliant with the government requirements about the preparation and use of JHAs [19].

To address these issues associated with the current way of producing and using JHAs in the construction industries, this study proposes an ontology-based automation approach to JHAs. More specifically, this paper proposes the schema of an ontological database for storing the JHA knowledge in the form of graph DB (aka knowledge graph). In this work, the development of the knowledge graph schema is conducted following a systematic ontological modeling methodology called METHONTOLOGY [20], and its detailed steps are described in this paper. Then, the performance of the developed ontological model is evaluated based on whether the knowledge graph database can provide an appropriate response to the queries about what type of risks are involved and how they should be mitigated, which are all essential questions to be answered when a JHA is performed.

Once the effectiveness of the proposed approach is validated, it is expected that, such a knowledge graph DB would be able to assist the practitioners with performing a more thorough and fully-compliant JHA in the job planning stage in a semi-automatic way. In other words, once this kind of system is available, field practitioners at construction sites can ask (“query”) the graph DB about what safety measures should be implemented for the specific construction activity under planning and receive help in generating a plan for the safe execution of the construction activity. Furthermore, it is conceivable that such a knowledge DB system can later be combined with a chatbot system and form a robotic assistant, which can help construction field engineers and work planners with safety hazard identification, risk assessment, and risk reduction in natural language communication with the chatbot.

The rest of this paper is structured as follows. In the following section, the most relevant previous works discussing an ontology-based approach related to hazard identification and risk assessment are reviewed, and the current knowledge gaps are identified. Then, in the method section, this paper’s main methodological framework, METHONTOLOGY, is explained. It is a systematic approach to developing an ontology, and this paper is organized following the processes of the methodology. The METHONTOLOGY process includes systematic validation of a proposed ontology, and therefore, the proposed ontological model of JHA is tested based on a number of queries pre-developed to show its effectiveness. Lastly, the discussion and conclusions section discusses the potential usefulness and future applications of ontology-based JHA.

## 2. Related Works

### 2.1. Ontologies for Construction Safety Management

Several previous researchers proposed an ontology-based knowledge base method to make construction safety management knowledge machine-readable. One of the seminal works in this area was conducted by Wang and Boukamp [12]. They identified semantic reasoning as one of the most suitable technologies to facilitate automated or semi-automated safety management in the construction domain. Based on that notion, they developed a framework to improve access to a company’s JHA knowledge by eliminating the complexity and time-consuming nature of traditional JHA processes. The proposed framework included a representation model and a reasoning mechanism. The representation model aimed to provide a systematic structure for modeling JHA knowledge in a machine-readable format, and the ontological reasoning mechanism was developed for identifying applicable safety rules from the knowledge base. In their proposed method, a combination of activity, tasks, and potential hazards constitutes the ‘condition’ for an unsafe scenario. Their work demonstrated that an ontology-based knowledge representation and reasoning technique could provide a shared understanding of the domain of construction safety management and a formal machine-readable model of the domain knowledge. Their work inspired other researchers to use similar approaches to sharing, reusing, and automatically processing construction safety management domain knowledge thereafter.

Based on a similar concept, Chi et al. [21] saw the possibility of utilizing construction safety management resources for generating JHAs with minimal human effort. They used an ontology-based text classification method to match safety measures identified from resources, such as safety guidelines, with unsafe scenarios. In addition, various document modification strategies, such as information retrieval techniques, were used to improve text classification effectiveness. This study demonstrated that it is possible to retrieve applicable safety measures information from existing documents using text mining techniques. As in the case of Wang and Boukamp’s [12] work, however, they used broadly-defined terminologies in classifying the factors affecting safety, such as ‘hazard’ and ‘safe approaches’. In addition, these did not consider exogenous factors affecting the occurrence of accidents, such as weather conditions or their interaction with the inherent characteristics of the activity.

Zhong and Li [22] also selected the ontology-based knowledge representation approach to model the knowledge about construction safety risks and proposed to implement the semantic inferring of construction accident causality based on the knowledge base. Specifically, they attempted to integrate knowledge about construction safety risks with knowledge about construction processes using a semantic inferring mechanism and tried to enhance the reusability of construction safety risks knowledge. However, their study was also limited in considering safety risks arising from temporary external conditions. In another work, Lu et al. [23] designed a meta-model for construction safety checking, which included categories such as ‘line of work’, ‘task’, ‘precursor’, ‘hazard’, and ‘solution’. According to their approach, when safety checking is needed, ‘precursors’ are appraised, and whether it will lead to an accident is determined. Their research showed a possibility to automatically reason about hazards and safety measures when given a construction activity, but it has limitations in assessing the varying possibilities of the actual occurrence of accidents and rather simply matches the precursors with hazards and “solutions”. Zhang et al. [18] also developed an ontology to formalize the knowledge about construction safety management. This ontology consists of the construction product model, process model, and construction safety model, and they tried to link the knowledge base with BIM software to visualize the inferred knowledge, such as hazardous areas and required safety management techniques, in a BIM environment. While this work suggests an advance in how to visualize safety information in a virtual construction site supported by BIM, it has limitations in modeling and expressing knowledge about detailed tasks, risks, and safety measures for different construction activities.

More recently, the ontologies of construction safety management knowledge started to be combined with machine learning techniques, such as natural language processing and image processing techniques. For example, Xiong et al. [24] introduced an ontology-based automated hazards identification system to evaluate job descriptions generated from site videos against the safety guidelines. Additionally, Zhong et al. [25] developed a process to identify potential construction hazards using an ontology-driven semantic approach to facilitate classifying construction images into categories automatically. These studies provided a novel approach to how the knowledge base model of construction safety management knowledge can be combined with or supported by other artificial intelligence approaches. However, the ontologies they used had limitations in modeling the subtle and more complicated, relationships between the task, risk factors, consequences, and safety measures, and rather focused on more obvious relationships, such as particular construction equipment and hazards associated with them.

Most recently, Jiang et al. [26] developed a knowledge graph to facilitate the analysis, querying, and sharing of the knowledge of construction safety management standards by analyzing the content of existing safety standards. The schema of the knowledge graph was developed based on 218 standards, and they found that the resultant knowledge graph allowed the retrieval of relevant construction safety management standards via natural language processing. This work provided another great example of how the knowledge of construction safety management guidelines and standards can be modeled into an ontology and how such a knowledge base can assist construction practitioners in finding relevant information about how to manage construction safety in a quick keyword search. However, the developed ontology was limited to standards, and therefore it does not have coverage over knowledge about the relationships among detailed tasks, safety risks, and how to mitigate the risks.

Table 1 provides a summary of previous studies most relevant to this paper including the above-mentioned papers.

### 2.2. Knowledge Gaps

As shown in Table 1, there have been a number of research papers proposing an ontology-based approach toward the automation of construction safety management. While these previous works have demonstrated the potential of automating the processes of identifying hazards and finding suitable safety measures based on an ontology, there were several factors that would limit the applicability of the ontologies in actual practice. First, most of the previous ontologies developed to represent construction safety management knowledge paid limited attention to the detailed causality of accidents, such as how hazards can turn into exposure, how exposure to a hazard can turn into an accident, and how such risks should be controlled. Therefore, the resulting ontologies would be capable of responding to simple queries such as “what hazardous conditions exist on the site?” or “where is the hazardous workspace located?” but not as effectively to more complex queries such as “Will the risk of primary hazard increase when the job is performed under a particular condition?” or “What are the most appropriate risk control methods when the job is performed under a particular condition”. Additionally, the previous ontologies focused on more obvious links between a job, hazards, and controls, such as the existence of fall risks when a worker works on height and the use of proper fall protection mechanisms, but they do not pay enough attention to more subtle factors that can contribute to the causation of accidents, such as particular weather conditions or site conditions. Site managers are required to consider such factors comprehensively and in a detailed way when preparing a JHA, and therefore the previous ontologies had limitations in fully assisting site managers with analyzing site hazards and devising effective safety measures.

It is conceivable that these limitations originated, at least partially, from the source of knowledge from which the ontology was developed. Table 1 shows that most previous research cases used documents containing construction standards, safety guidelines, and regulations (e.g., OSHA regulations, building codes, and construction manuals). While these are important resources useful for identifying the general knowledge of standard methods for managing safety risks, they may provide limited information about detailed construction processes/methods, the hazards/risks associated with them, and detailed measures to control the risks most suitable under particular circumstances. Often, knowledge about such detailed technical matters exists only in the form of implicit knowledge, meaning it has not been formalized yet and is more difficult to verbalize or write it down. Assessing a safety risk given the characteristics of a specific process and the environmental conditions and proposing a practicable solution to mitigate the safety risk cannot be performed without one’s experiential knowledge about what could happen and what should be conducted. Such a type of knowledge is required in preparing an effective JHA, and therefore the knowledge base developed to assist that very process must represent more specific, more detailed, more experiential knowledge about what to do in each step to ensure safety than the content of safety management authorities’ guidelines or regulations statements. It is highly likely that such detailed how-to-manage-risks knowledge exists implicitly, either in practitioners’ heads or in JHA documents they previously developed, but the utilization of these sources in developing an ontology was very limited in previous research efforts. This research has been conducted with the aim of addressing these knowledge gaps existing in the current body of knowledge.

## 3. Methodology

According to D’Avanzo et al. [27], manual ontology construction enables the insertion of meaningful information into an ontological system and involves the creation of concepts taxonomy with the direction of human expertise. A comprehensive ontology development methodology that can guide and manage the development process is critical for its quality as the utility completely depends on this development method [28]. Therefore, an ontology development methodology should comprise a set of well-established principles, processes, practices, methods, and activities used to design, construct, evaluate, and deploy ontologies [29].

This research was conducted using one of the leading manual ontology engineering methods called METHONTOLOGY. The process starts with the specification phase (1), which involves defining the purpose, scope, domain, and requirements of the development guided by competency questions. The knowledge acquisition phase (2) is about acquiring knowledge from experts, written knowledge sources, figures, and previous ontologies in conjunction with knowledge acquisition techniques such as brainstorming, interviews, and text analysis. Knowledge acquisition starts from the specification phase and decreases with the progress of the ontology development process. The conceptualization phase (3) is about structuring the domain knowledge in a conceptual model using the glossary of terms that represent the domain knowledge and its meanings. The integration phase (4) aims to speed up the ontology development process by integrating other compatible ontologies instead of starting from scratch. The implementation phase (5) requires an ontology to be implemented as an accessible database; the result of this phase is a codified ontology written in a formal ontological language. Finally, the evaluation phase (6), also known as the verification and validation phase, is about evaluating the developed ontology against the goal of the ontology [20].

Out of the several possible approaches available in developing a class hierarchy, this paper has adopted the top-down approach. This is a manual approach carried out with the involvement of domain experts [30]. The top-down development process starts with the definition of the most general concepts in the domain and subsequent specialization of the concepts [31], thereby top-down. Therefore, the analysis started from the explicit concepts presented in the JHA documents, and the identification of implicit concepts and relationships was done subsequently. During the first phase of analysis, information provided under the explicit concepts was analyzed, and implicit concepts were reasoned from the explicit concepts. During the second phase of analysis, both explicit and implicit concepts identified were analyzed together, creating an exhaustive list of concepts necessary for performing a JHA.

## 4. Development of the Ontology for Job Hazard Analysis Knowledge Graph

**Figure 1:**
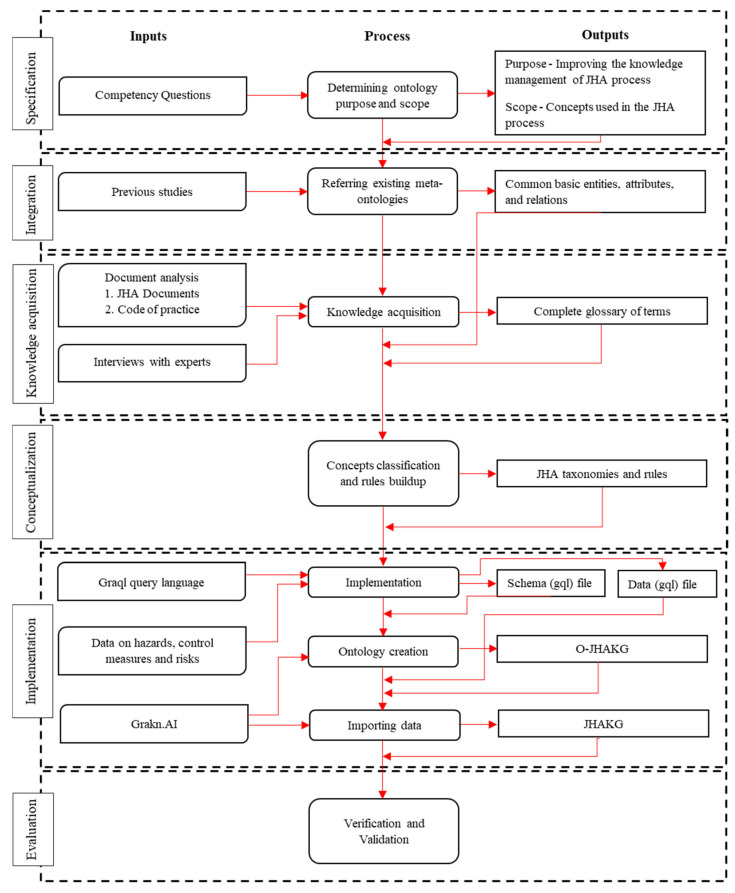
Development process of JHAKG.

This section describes how the METHONTOLOGY steps were applied in the process of developing the Ontology for Job Hazard Analysis Knowledge Graph (O-JHAKG). Figure 1 overviews the overall development process with inputs, process, and outputs involved at each phase. The following subsections provide a detailed description of each phase this research followed.

### 4.1. Phase 1: Specification

According to Fernandez et al. [20], the development of an ontology should not be started without clearly setting its purpose and scope. The scope limits the ontology, specifying what must be incorporated into the ontology and what must not. It is a crucial step for minimizing the amount of data and concepts to be analyzed [32]. Specifying the Competency Questions (CQs) in the specification phase is important in determining the scope of the ontology. CQs include a set of questions that the ontology should be capable of answering. Furthermore, the CQs are used in the validation of the ontology by checking if the ontology can provide answers to the questions (i.e., queries) [33]. It is recommended that a competency question be related to each concept in the ontology [32].

#### 4.1.1. Purpose and Domain

O-JHAKG is a domain ontology to support the JHA process in the construction industry with the primary purpose of automating the integration, reasoning, and searching of the hazards and risk information related to the JHA process. Hence, the ontology should only incorporate the concepts used in the JHA process. The resultant JHAKG should be able to act as a knowledge base that the JHA team can query and obtain specific information about hazards, risks, and control measures involved with the job they are planning.

#### 4.1.2. Requirements

In this research, the requirements for O-JHAKG were identified through consultation with 18 construction domain experts experienced in the JHA process. (A detailed discussion of the background and qualifications of these interviewees is elaborated in Section 4.3.2, Expert Interviews). The experts indicated that the risk levels of hazards are influenced by external factors and therefore such factors should be considered in the JHA process. Examples of such external factors are weather conditions, workplace conditions, and atmospheric conditions, and they affect construction activities and often increase the risk involved with hazards. Safety personnel often use their implicit knowledge to identify these changes in safety risks and reflect that in JHA outputs. Thus, O-JHAKG should include the ontological elements that can represent such implicit knowledge of safety personnel.

As a next step, CQs were developed based on the result of JHA document analysis and expert interviews. The CQs that cover the main concepts of O-JHAKG are listed in Table 2. The CQs mainly concern (1) the identification of primary hazards, (2) the identification of control measures for primary hazards, (3) the identification of secondary hazards and control measures, and (4) the identification of changes in risk levels due to external factors.

### 4.2. Phase 2: Integration

Reusing existing ontologies and meta-models instead of starting from scratch can speed up the ontology development process, so it is recommended in METHONTOLOGY. Therefore, the previous ontologies and meta-models relating to the identification of hazards and control measures were reviewed in this research. The previous ontologies listed in Table 1 included the definition of broad concepts such as “hazards” and “control measures” and their relations, and therefore O-JHAKG referred to these previous ontologies. However, the detailed relations between external factors and safety risks, and between risk levels and appropriate control measures, were found not defined in the previous ontologies, and therefore these ontological elements were identified as needed to be developed new. The ontologies developed by Lu et al. [23] included concepts such as the conditions, events, and sequences (“precursors”) that preceded an accident, and although these elements are not fully aligned with O-JHAKG, the concepts embedded in their ontology, such as work team, physical system, and environment, were used in this research to identify implicit knowledge used in JHA processes.

### 4.3. Phase 3: Knowledge Acquisition

#### 4.3.1. Document Analysis

JHA forms collected from 10 different contractors who are engaging in water facility construction and maintenance work in South Australia were used to extract knowledge related to hazards, risk factors, and control measures. In total, 115 JHA cases (i.e., JHA documents) analyzing 22 construction activities were analyzed to design O-JHAKG. The JHA documents used in this research included the most common construction activities, such as excavation, concreting, welding, compaction, plumbing, drilling, and lifting.

Additionally, Australian Code of Practice documents, which provide practical guides to achieving the standards of health and safety requirements under the Australian legal framework [34], were used to extract knowledge about how to manage health and safety (H&S) risks in construction works according to the requirements of the government authorities regulating industrial H&S matters. In total, 12 Australian Code of Practice documents were analyzed to design O-JHAKG, including *Model Codes of Practice: Excavation Work* [35], *Model Codes of Practice: Hazardous Manual Tasks* [36], *Model Codes of Practice: Welding Processes* [37], and *Model Code of Practice: Abrasive Blasting* [38], as they are related to construction works. The analysis of the Code of Practice documents helped with defining the concepts used in the JHA knowledge domain and with understanding the potential relationships in the O-JHAKG.

**Figure 2:**
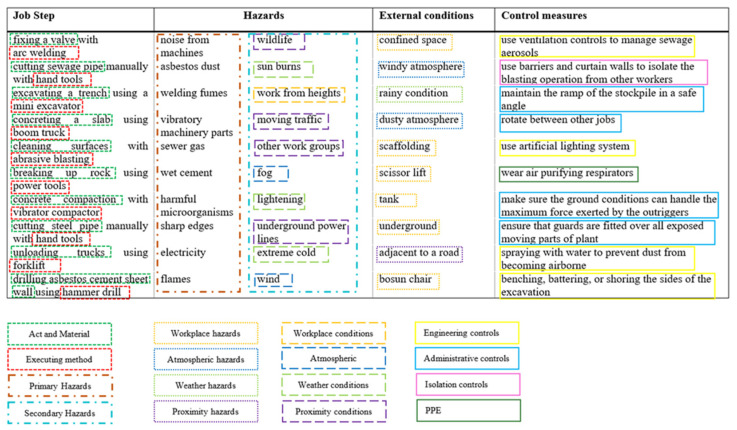
Example of the glossary of terms extracted from JHA documents and the classification of concepts.

From the document analysis based on the JHA forms and the Code of Practice documents, a complete glossary of terms for the JHA knowledge domain was produced, and the glossary was used to classify the concepts and build their relationships. Since one of the main requirements of JHAKG is to evaluate the risk of hazards, it was required to identify the factors contributing to the risk. As the collected JHA documents included multiple cases of analyzing the same type of hazard, the document analysis allowed the comparison of the risks related to the same hazard under different conditions. Specifically, the comparative analysis of JHA documents revealed that there are several external conditions affecting the risk (i.e., the chance or anticipated severity of an accident) implied in the JHA documents. As a result, the external factors identified also entered the glossary. Figure 2 visualizes a snippet of the glossary of terms and how the classification of main concepts was performed in the document analysis. A more detailed explanation of the classification system used in this research is provided in Section 4.4, Conceptualization section.

#### 4.3.2. Expert Interviews

Three rounds of interviews were conducted with the same set of experts who have participated in the ontology requirement identification phase to identify the concepts and the relationships to be included in O-JHAKG. Those 18 experts were experienced construction professionals in the Australian construction industry and were selected through purposive sampling. The information on the background and qualifications of the participants is given in Table 3. As shown in the table, the experience of the participants ranged from 5–34 years, with an average of 20 years. Out of the total interviewees, 56% had been directly involved in the JHA process as their day-to-day work, while the remaining 44% had only indirect involvement in the JHA process (The involvement of each interviewee in the JHA process has been identified in Table 3 with a ✓ symbol). The indirect way of becoming involved in the JHA process includes consultation, monitoring, reviewing the JHA documents, and providing training on JHA. Therefore, the sample represented construction professionals who have considerable experience and knowledge about the JHA process including hazard identification and risk analysis.

The first round of expert interviews was organized to validate the findings of the document analysis and to extract the implicit knowledge of safety personnel. For these interviews, an interview guideline was prepared so that the interviewees could present their ideas on the findings of the document analysis and validate the concepts identified in the initial phase. Furthermore, they were asked to categorize the concepts based on their frame of knowledge about hazards and risk management so that the resulting ontology’s reasoning capacity could be increased. The interview guideline also included questions about the implicit concepts identified through the comparative analysis between JHA cases about the same type of hazards. Additionally, some open-ended questions were included regarding other implicit risk factors that should be considered when developing a JHA document. Further questions were also directed to identify the subtle relationships between direct causes, external factors, and the consequence and probability of accidents. Therefore, the interview data collected in the first round helped to confirm that there are indirect or external conditions contributing to the risk implied in the JHA documents.

During the second round of the interview, the analysis results of the previous interview round were presented to the participants, and their feedback was collected. The third round of interviews involved presenting a list of statements to the interviewees, which described the structure of O-JHAKG. This was conducted to validate the final concepts and relationships that represented the domain knowledge of JHA. Hence, the information collected from the three rounds of interviews enabled O-JHAKG to include the ontological elements that could not be identified through document analysis due to their implicit nature.

### 4.4. Phase 4: Conceptualization

The process of conceptualization is an essential aspect of developing an ontology, as it involves the identification and definition of the concepts and relationships that are intended to be incorporated into the ontology. Therefore, the data collected from JHA documents and interviews were thematically analyzed to recognize significant concepts utilized by safety personnel in the JHA process. The coding and categorizing of data were iteratively conducted by moving back and forth over the data until a sense of understanding or interpretation was reached. In addition, the previous ontologies and accident causation theories provided some guidance for the analysis. This analysis revealed how each of these concepts contributed towards the process of risk evaluation. It identified the important attributes of entities that play a critical role on JHA domain knowledge reasoning. The detailed analysis of the qualitative data collected through the interviews which shows how each safety concept was analyzed to obtain the other concepts is not included in this paper as it is outside of the scope of this paper.

**Figure 3:**
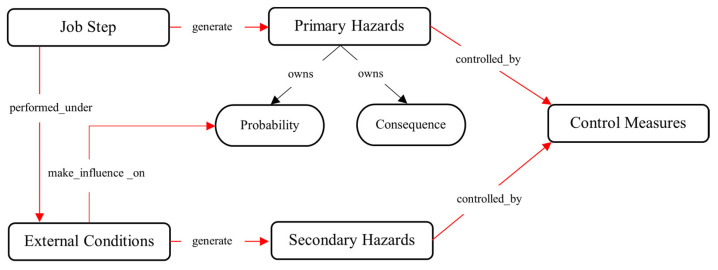
High-level concepts and links of O-JHAKG.

Figure 3 shows a high-level overview of the top-level concepts of O-JHAKG. A job step (e.g., concrete pouring, drilling the ceiling panels, or cutting steel frames) performed under certain circumstances (e.g., tools/equipment used for the job, workplace conditions, and weather) is associated with some hazards: (job step)—*generate* → (primary hazard). Following the previous ontologies and the relevant literature, a hazard is defined as the potential to harm a person. A risk concept is involved here; a hazard has a “potential” to harm a person, and therefore it might or might not turn into an accident depending on the degree of exposure to the hazard and other conditions affecting the causation of accidents [39]. Additionally, different types of hazards can lead to accidents of different levels of severity. Therefore, the risk of a hazard is determined by two factors, probability and consequence (i.e., severity) [39]. In the current ontology, such a relationship between a hazard and the level of risk is formalized in the following links: (hazard)—*owns* → (probability) and (hazard)—*owns* → (consequence). Then, some external conditions under which the job is performed (i.e., (job step)—*performed under* → (external conditions)) can affect the probability of an accident, as represented in the following link: (external conditions)—*make influence on* → (probability). Apart from the hazards that are associated with a job step inherently (“primary hazard”), there are hazards that are solely associated with external conditions, such as dusty environments and cold weather, namely “secondary hazards”. Therefore, another link is formalized as the following link: (external conditions)—*generate* → (secondary hazards). The primary hazards and secondary hazards are controlled by control measures; thereby, the following links are formalized: (primary hazards)—*controlled by* → (control measures) and (secondary hazards)—*controlled by* → (control measures). As the developed JHAKG only performs a risk analysis for primary hazards, no links were created in Figure 3 for secondary hazards with probability and consequence. The following subsections provide a description of the detailed concepts that come under these top-level concepts, their classifications, and the links between them.

#### 4.4.1. Sub-Concepts of Job Steps

**Figure 4:**
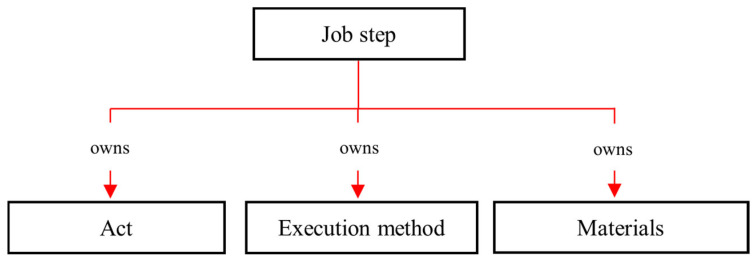
The sub-concepts of *job step*.

The analysis of JHA documents revealed the characteristics of job steps and their different relationships with hazards. In JHA documents, job steps are normally expressed as a combination of (i) act (e.g., excavating, welding, painting, cutting, and compacting), (ii) execution method (e.g., mini excavator, gas welding, spray painting, oxy-acetylene torch, and rammer), and sometimes (iii) the materials associated with the job step (e.g., asbestos sheet, sewage water pipe, and socket outlet). All these three elements of the job step can relate to a particular hazard. Following this, in O-JHAKG, the *job step* concept is expressed as a combination of an *act, executing methodology*, and *the associated material*: (job step)—*owns* → (act), (job step)—*owns* → (execution method), (job step)—*owns* → (materials). Therefore, as shown in Figure 4, the taxonomy of a *job step* includes the concepts of act, execution method, associated material, and external conditions.

#### 4.4.2. Sub-Concepts of External Conditions

**Figure 5:**
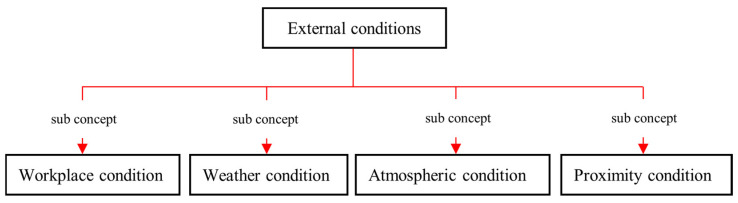
The sub-concepts of *external conditions*.

Construction workers perform their duties under different conditions. Those conditions can be broadly divided into four main categories namely: workplace, proximity, weather, and atmosphere as illustrated in Figure 5. Other than the hazards resulted as a direct execution of job steps, these conditions can also create hazards to the workers. Thus, the JHA process needs to consider these *external conditions* as well to get an overall picture of the hazards that can impact the workers. Moreover, these *external conditions* can create influences on the risk of hazards by altering the probability (this is further explained in Section 4.4.5, under “Rules Related to Risk Evaluation”). Thus, incorporating the concepts of *external conditions* is vital for the performance and the comprehensiveness of JHAKG.

#### 4.4.3. Sub-Concepts of Hazards

The analysis of different JHA documents analyzing similar activities revealed important characteristics of the hazards discussed in the JHA. Even if the activity and its job steps are identical, the list of hazards may not be identical. The hazards that are common across the JHA cases would be the ones directly related to the inherent characteristics of the job steps, namely “primary hazards”. On the other hand, the hazards that are not common across the JHA cases (even though the activity and job steps are the same) would be related to a specific external condition, and these types of hazards can be labeled as “secondary hazards”.

**Figure 6:**
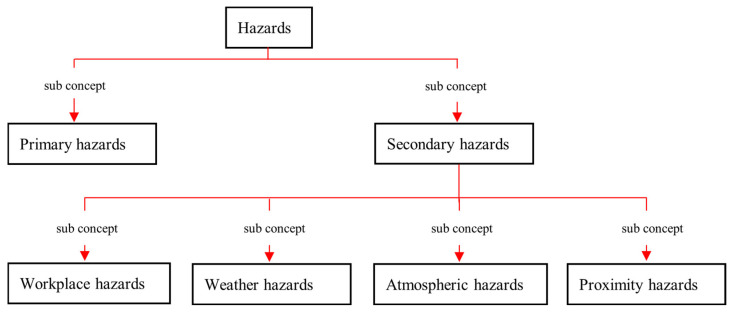
The sub-concepts of *hazards*.

The secondary hazards can be further divided into subgroups depending on the cause, as shown in Figure 6. The following are the description of these sub-concepts of secondary hazards.

1. Proximity hazards—Hazards that arise from the proximities (e.g., underground service lines, mobile plants, other work groups, public)
2. Workplace hazards—Hazards that arise due to the nature of the workplace (e.g., falls, lack of oxygen, bush fire, wild animals, slips)
3. Weather hazards—Hazards that arise from the existing weather condition (e.g., lightening, UV rays, heat stress, extreme cold)
4. Atmospheric hazards—Hazards that arise due to the existing atmospheric condition (e.g., dust, flammable atmosphere, contaminated atmosphere, lack of visibility)

#### 4.4.4. Sub-Concepts of Control Measures

**Figure 7:**
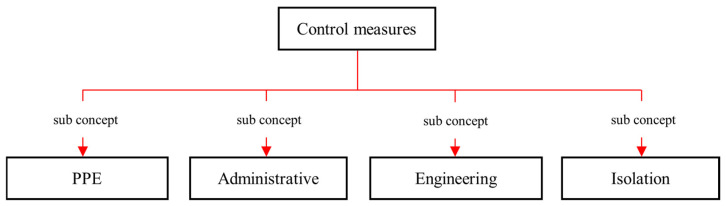
The sub-concepts of *control measures*.

Authoritative sources of risk management principles explain that control measures are categorized into six categories such as elimination, substitution, isolation, engineering controls, administrative controls, and personal protective equipment (PPE) [40,41]. The JHA document analysis revealed that out of the six categories, JHA considers only the last four types of control measures. It is because the first two types of risk control measures, elimination and substitution, can be considered only in the early stages of the construction project when construction methods are determined. Therefore, when it comes to the construction phase, choosing an elimination or substitution type of control measure is nearly impossible as it would require a major change in the whole process of construction or in the construction design. Therefore, it is more plausible to consider a control measure among the last four types, and therefore O-JHAKG includes these four types of control measures as the sub-concepts of *control measures*, as shown in Figure 7. The formalization of the type of control measures in this way allows the JHA results to clearly indicate the type of control measures, thereby the power of control.

#### 4.4.5. Rules Related to Risk Evaluation

**Figure 8:**
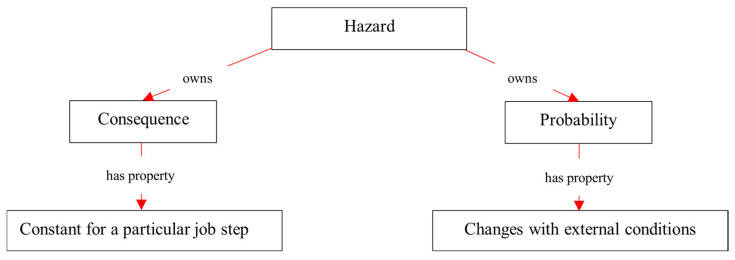
Risk variables of *hazards*.

As explained previously, the risk of a hazard is related to two concepts, probability and consequence. In O-JHAKG, the consequence of a hazard is defined as a constant characteristic, meaning the consequence is the worst possible result of the accident originating from the hazard. Thus, the consequence would not be affected by external conditions when the job step is the same. On the other hand, the probability of a hazard (meaning the probability that an accident occurs due to the hazard) can be changed by external conditions under which the job step is executed. For example, the probability of an asphyxiation accident is higher when welding is performed in a confined workplace than in an open environment, and thereby an increased risk. These relationships between the risk of hazard and external conditions were identified through document analysis and expert interviews, and they are coded as a rule as part of O-JHAKG, as shown in Figure 8. This means that certain relationships between some external conditions and the probability variable for some hazards can be entered in the form of a rule in JHAKG (as discussed in the implementation section, the graph database management system used for this research allows this kind of formalization).

### 4.5. Phase 5: Implementation

**Figure 9:**
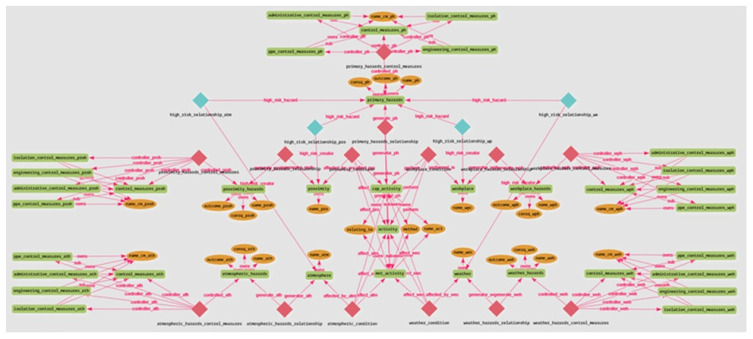
Visualization of O-JHAKG implemented in Grakn.

The proposed ontology for JHAKG was implemented using the GRAKN.AI graph database management system, a deductive database for artificial intelligence. GRAKN.AI provides a suitable development environment for a knowledge graph, allowing complex data modeling, verification, scaling, querying, and analysis [42]. A database generated within GRAKN.AI uses an ontology to enable the modeling process of highly complex datasets, operating as a data schema constraint to assure information consistency [43]. GRAKN.AI comprises two materials: the storage (Grakn) and the query language (Graql). Graql is a declarative, knowledge-oriented graph query language that allows concepts and relationships to be categorized into distinct types, enabling automatic reasoning over the represented knowledge [42]. Using Graql, the JHA domain concepts and their links identified above were written into an operable knowledge database schema, resulting in a schema file. The schema file, therefore, contains the blueprint of the JHAKG and provides an underlying structure to store highly interconnected JHA domain concepts. Figure 9 shows a visualization of O-JHAKG in the Grakn Workbase once the schema file is uploaded into the Grakn via its console.

#### 4.5.1. Importing Data

GRAKN.AI stores data so that computers can process the meaning of information in the complete context of their relationships. Consequently, the semantic layer of Grakn enables the processing of complicated information more intelligently, with minimum human intervention [43]. The data extracted from the JHA documents and Code of Practice documents under the concepts of *job steps*, *primary hazards*, *secondary hazards*, *external conditions*, and *control measures* were hand coded in Graql using a source code editor. The coding process was executed in a way that all data maintain the relationships included in the knowledge graph schema. The resultant data file was imported into the O-JHAKG via the Grakn console and the JHAKG was created.

#### 4.5.2. Knowledge Graph Reasoning and Queries

Once a JHAKG is populated, the knowledge graph can respond to queries related to primary hazards, secondary hazards, initial risks, and control measures. Specifically, queries can infer types, relations, context, and pattern combinations. In Grakn, such queries are written using Graql, which translates a query into its logical equivalents and analyzes them against the database.

Grakn supports two types of inference mechanisms. The first one is *type inference*. It is based on the semantics as defined in the knowledge graph schema, and it is the one that helps to extract the information for simple queries relating to JHA.

The second one is *rule-based inference* that involves if-then rules defined by expressions of the following form:

left-hand-side of the rule (body) → right-hand-side of the rule (head)

where the head and body are a pair of Graql patterns. The body is also known as the antecedent, while the head is known as the consequent. Consequently, the rules are statements of the following form:

q_1_ ∧ q_2_ ∧ … ∧ q_n_ → p

where q and p are atoms, each of which corresponds to a single Graql statement and “∧” denotes the conjunction syntax, and “→” denotes implication. Whenever the left-hand-side pattern is found in the data, the right-hand-side pattern can be assumed to exist and be optionally materialized (inserted).

The information extracted from the JHA documents and expert interviews on risk evaluation was inserted into the schema file as if-then rules to facilitate rule-based inferencing. Rules are a powerful tool in Grakn that allow users to reason over the explicit relationships between data and infer implied knowledge at the run-time. It enables the users to dramatically shorten the length of complex queries and perform explainable knowledge discovery. In the context of this research, this function can be used to reason over the weather conditions, workplace conditions, atmospheric conditions, and proximity conditions against primary hazards in determining the risk level. In other words, the rules can evaluate the hazards against the external conditions presented at the execution of a job step and infer whether the risk can be increased or not. The development of these rules was supported by the implicit knowledge of the experts acquired through interviews because such knowledge is difficult to extract from the source of explicit knowledge. Such rules representing implicit knowledge, therefore, can be viewed as a novelty in JHAKG and make it distinct from the previous ontologies.

Table 4 shows several examples of rules that were recorded in O-JHAKG with their meaning in natural language. The rules were categorized based on the nature of the external conditions that influence the risk level of primary hazards. Accordingly, four types of rules were developed as “Workplace-primary hazards risk rule”, “Weather-primary hazards risk rule”, “Atmosphere-primary hazards risk rule”, and “Proximity-primary hazards risk rule”. Each type of rule pertains to a different external condition that influences the risk level of primary hazards in the workplace. An explanation for each type of rule was provided in the table, along with a Graql code example for implementing the rule.

### 4.6. Phase 6: Evaluation

Ontology evaluation can be defined as “a technical judgment of the content of the ontology with respect to a frame of reference during every phase and between phases of their life cycle” [44]. This section describes the efforts for evaluating the developed O-JHAKG, including the verification (Section 4.6.1) and validation processes (Section 4.6.2).

#### 4.6.1. Verification

Ontology verification is defined as “the ontology evaluation which compares the ontology against the ontology specification document, thus ensuring that the ontology is built correctly” [45]. For a newly developed ontology, such as O-JHAKG, asking competency questions and consulting experts are the most common methods to verify the semantics of the ontology [33]. Furthermore, the built-in reasoner of Grakn was used in both the O-JHAKG creation and implementation stages to check the logical structure of the data to ensure that the codes were written correctly [42].

In this phase, the CQs listed in Table 2 were translated into Graql queries. Example queries instantiated are: (1) What are the hazards that can have a high severity during the execution of “concreting using a boom truck”? (2) What are the hazards that can lead to “breathing difficulties”? (3) What are the different types of control measures to control the “falling from ladder” hazard? (4) What are the different construction methods available for the “ground compaction” work, and what are the common hazards associated with the work? Sample data, created by the authors for verification purposes only, were used during the ontology development phase for self-checking purposes [46]. The test on such simulated data verified the semantic correctness of the ontology under development. Additionally, when the initial development process was complete, O-JHAKG was presented to the experts who participated in the semi-structured interviews, and they reviewed each concept and relation included in the ontology and confirmed semantic correctness.

#### 4.6.2. Validation

Lastly, a hypothetical case study was performed to demonstrate the correctness of JHA outputs generated from the JHAKG database and the usefulness of the proposed approach. The hypothetical case study was a water infrastructure construction project performed in Adelaide, South Australia. The project comprises a new water treatment plant, distribution system, and many enhancement activities to the existing facilities. Thus, the project includes dynamic working conditions and numerous high-risk construction activities such as excavations deeper than 1.5 m, diving activities, asbestos-related activities, demolition of load-bearing structures, etc.

Specifically, the case study was performed assuming two scenarios. In Scenario 1, the interest of the test is in ensuring JHAKG’s functionality of searching for hazards information for different job steps according to their executing methods and different external conditions (workplace, atmospheric, proximity, and weather conditions) under which they are performed, and the different types of control measures with their respective category in the hierarchy of control measures and risk information of hazards. In Scenario 2, the interest of the test is in ensuring the JHAKG’s capability of reasoning risk levels as affected by external conditions.

Scenario 1: The JHA team wants to identify the hazards associated with an arc welding activity and the hazards associated with the work environment underground, and then they also want to identify the appropriate risk control measures for identified hazards. In this scenario, the construction work planner would want to query the JHAKG database in three steps as the following.

- Query 1: What are the hazards that can arise from the execution of an arc welding activity, and what are the consequence levels and possible outcomes caused by the hazards?

**Figure 10:**
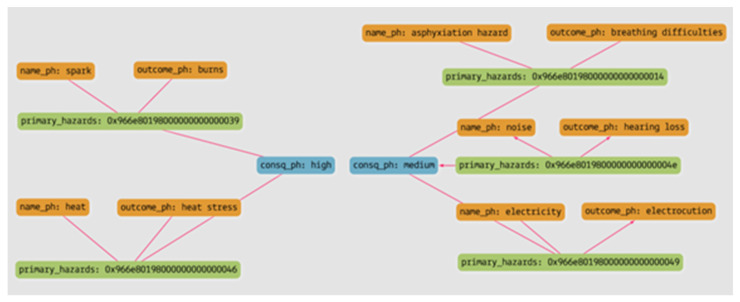
The result of Query 1, in a graph form.

Figure 10 and Table 5 show the result of the query in the form of a graph and tabular form. As shown in the figure, the knowledge graph was able to extract five different hazards associated with the arc welding process and to retrieve their outcomes with consequence levels. Therefore, safety personnel can prioritize the hazards and treat them according to their consequence level.

- Query 2: What are the hazards related to external conditions and the consequence level and possible outcome caused by the hazards?

**Figure 11:**
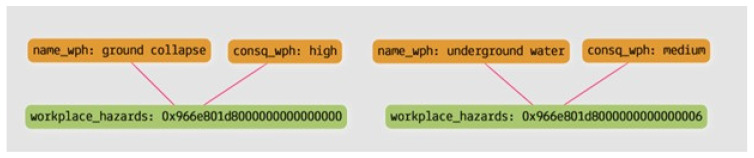
The result of Query 2, in a graph form.

Figure 11 and Table 6 show the result of the query in the form of a graph and tabular form. JHAKG can retrieve not only the hazards associated with the job steps but also the hazards that are associated with the external conditions present at the moment of job-step execution. Since the knowledge graph can identify the consequence levels of each hazard, control measures can be implemented according to their priority level.

- Query 3: What are the control measures to mitigate the impact of unstable surfaces and edges when the hazard has a high consequence level, and what are their respective categories?

**Figure 12:**
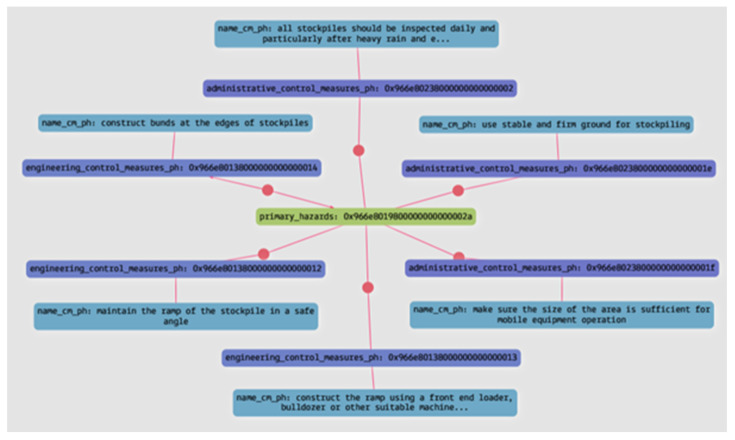
The result of Query 3, in a graph form.

The control measures to be implemented for a hazard will vary depending on its level of consequences. JHAKG can accurately identify the necessary control measures by considering both the hazard type and its consequence level. This query demonstrates JHAKG’s ability to list various control measures, enabling construction planners to select the most appropriate measure for a given hazard based on their assigned effectiveness category. Figure 12 and Table 7 show the result of the query in the form of a graph and in tabular form.

Scenario 2: The JHA team needs to identify the changes in the risk level of primary hazards due to the external conditions at present. Thanks to the risk evaluation rules included in a O-JHAKG, a JHAKG contains knowledge about whether external conditions, such as workplace conditions, proximities, atmospheric conditions, and weather conditions, would increase the risk level (by affecting the probability of occurrence of accidents). Under these circumstances, the construction planner can query the JHAKG database, such as the following.

- Query 4: What are the hazards that can have a high risk when arc welding is performed in a manhole, and what are the control measures to mitigate the risk of the hazards and the control measures for the hazards related to the working environment?

**Figure 13:**
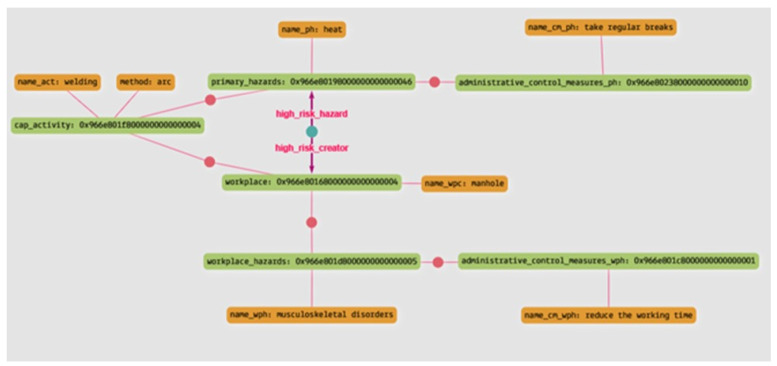
The result of Query 4, in a graph form.

The result of this query indicates that when arc welding is performed in a manhole, the risk of heat can be increased due to the enclosed nature of the work environment. The arrows starting from the green dot in Figure 13 indicate a high-level risk. Additionally, this query returns the control measures to mitigate this high risk of heat stress and the control measures to mitigate the risk arising from the workplace characteristics in their respective category. Table 8 show the results of the query in the tabular form.

## 5. Discussion and Conclusions

JHA is an essential element of safety management systems in construction, involved with understanding the detailed tasks for each construction activity, identifying hazards, assessing risks associated with the hazards, and developing plans regarding how to mitigate the risks. In the current practice, however, JHA is performed manually, relying on the safety personnel’s experiential knowledge and observations. There were several ontologies developed to formalize the JHA knowledge domain, but previous ontologies were limited in representing the detailed relationships between job steps, hazards, risks, and the influence of external factors on the risks, and, therefore, they did not provide the sufficient coverage and depth of knowledge required to automate JHA processes based on a knowledge base.

This research was conducted to create a new ontology that comprehensively represents the JHA knowledge domain, including the implicit knowledge about safety risks and control measures construction work planners and safety managers use when developing JHA documents. Specifically, 115 actual JHA documents and interviews with 18 JHA domain experts were analyzed and used as the source of knowledge for creating a new JHA knowledge base, namely the Job Hazard Analysis Knowledge Graph (JHAKG). To ensure the quality of the developed ontology, a systematic approach to ontology development called METHONTOLOGY was used in this process. The METHONTOLOGY process includes specifying the goal of ontology development, referring to previous ontologies, acquiring knowledge sources, developing the hierarchical structure of concepts and their semantic relationships, implementing the developed ontology into an operable database instance, and evaluating the performance of the ontological database against the performance goals (e.g., competency questions) that were determined at the initial stage of development. The output of this research (i.e., an ontology representing JHA knowledge) provides a framework for operating JHA information automatically in a knowledge base system and possibly generating JHA documents automatically. Such an ontology representing the detailed relationship between the information contained in JHA documents, including implicit elements considered in the JHA process, has not been developed in previous works to the best knowledge of the authors, and in that regard, the presented research work has some novelty.

The case study performed for validation purposes demonstrates that a JHAKG can operate as a knowledge base that a construction work planner can query regarding the types of hazards inherently associated with the job steps or the external factors under which the job is performed, the level of risks, and appropriate control measures to mitigate the risks. As the JHAKG is a database of knowledge representing a large number of actual JHA cases previously developed and also implicit knowledge that has not been formalized in any explicit forms yet, the quality of JHA documents produced from queries to the database is expectedly higher than the ones produced by an individual safety manager in terms of completeness and comprehensiveness. In this regard, the JHAKG can be seen as a first step toward the data-based automation of construction hazard and risk management.

As O-JHAKG was developed as a general framework for containing JHA knowledge, it is conceivable that many versions of JHAKGs can be populated based on the proposed ontology, creating a comprehensive knowledge base of industrial hazards and risks at the level of a company, an industry, a region, or a country. Once such a JHAKG is populated with sufficient inputs from previous JHA cases, accident cases, and inputs from safety management experts, the knowledge base can assist practitioners in industries with preparing appropriate JHAs, which can result in a reduction in the number of accidents.

Although the context in which the research was conducted was construction projects, the idea put forward in this paper would be applicable to other industries as well. According to the US’s Occupational Safety and Health Administration (OSHA), a JHA should be conducted in any jobs that involve the potential to cause severe or disabling injuries or illness. The O-JHAKG developed in this research would be applicable to JHAs developed in industries other than construction because the JHA process and information required in the form would be the same as it is in the construction industry.

Another foreseeable possibility is to combine chatbot technology with a JHAKG. Recently, chatbot applications such as ChatGPT are drawing significant attention from all directions and suggest a near future where people can ask a chatbot complex questions and get an answer in natural language with a minimal time lag. Such a chatbot’s industrial application is discussed in various industries, including construction. Therefore, it would not be far-fetched if we imagined a near future where a construction work planner would ask a chatbot safety management questions such as “what are the risk items for the job planned for next week, and what needs to be done to ensure safety”?

In this research, the primary sources of knowledge utilized for developing a JHAKG were twofold, the previous JHA documents and the interview data with experts. However, there are other important sources of knowledge for JHA matters this research paid limited attention to, such as accident case databases. Therefore, future research is needed regarding how other knowledge sources can contribute to JHAKG so that the knowledge base can be even more comprehensive. Furthermore, in preparation for integration with a chatbot system, as described above, how natural languages rather than graph DB query languages can be used to query the knowledge base and obtain all the relevant information about hazard and risk management can be further studied.

## Figures and Tables

**Table 1:** Comparison of existing ontologies for construction safety management knowledge.

Authors	Ontology Modeling Method	Sources of Knowledge Used to Construct the Ontology	Purpose of the Ontology
Jiang et al. [26]	Manually matching, classifying, and filtering entities with concepts in the ontology and encoding them in a machine-readable format	National, industry, local, and corporate construction safety standards	An ontology to facilitate the modeling of construction safety standards knowledge
Zhong et al. [25]	Using Natural Language Processing (NLP) to match annotated images with the ontology semantically	Chinese specification Quality and Safety Inspection Guide of Urban Rail Transit Engineering	An ontology to model the hazard events
Xiong et al. [24]	Manually encoding regulatory documents in a machine-readable format	Safety Handbook for Construction Site Workers The Construction (Design and Management) Regulations 2015 Recommended Practices for Safety and Health Programs in Construction	Construction safety ontology to assist the evaluation process of operation descriptions generated from site videos against safety guidelines extracted from the documents
Zhang et al. [18]	Manually encoding construction safety regulations and industry safety best practice reports in a machine-readable format	OSHA regulation 1926 Occupational Injury and Illness Classification Manual Construction Solutions Database	Construction safety ontology to formalize the current construction safety knowledge and to support safety hazard identification and mitigation through BIM
Zhong and Li [22]	Manually encoding risk information in a machine-readable format	Building technical codes or regulations Construction manuals Best-practice construction rules or experts’ experience literature	A meta-ontology model to integrate the risk knowledge domain with the risk monitor object domain.
Lu et al. [23]	Manually encoding construction safety checking information in a machine-readable format	CPWR construction solution database OSHA regulations	An ontology for automated construction safety checking
Chi et al. [21]	A semi-automated procedure based on text classification	Center to Protect Workers’ Rights (CPWR) construction solution database NIOSH FACE reports OSHA standards	A construction safety domain ontology that allows users to locate a specific activity and hazard and then retrieve possible solutions to support the JHA
Wang and Boukamp [12]	Manually encoding JHA information in a machine-readable format	Occupational Injury and Illness Classification Manual MasterFormat 2004 Edition from Construction Specifications Institute JHA documents	Concept ontology to assist the JHA process

**Table 2:** Competency Questions.

Main Requirements	CQ ID	CQ Text
Identification of primary hazards	CQ 1	What are the primary hazards of a job step?
Identification of control measures for primary hazards	CQ 2	What are the control measures of a primary hazard?
Identification of secondary hazards and control measures for them	CQ 3	What are the workplace hazards and control measures when a job step is performed on a particular workplace?
	CQ 4	What are the weather hazards and control measures when a job step is performed in a particular weather condition?
	CQ 5	What are the proximity hazards and control measures when a job step is performed in a particular proximity?
	CQ 6	What are the atmospheric hazards and control measures when a job step is performed in a particular atmosphere?
Identification of changes in risk levels	CQ 7	Will the initial risk of primary hazards increase when a job step is performed in a particular workplace?
	CQ 8	Will the initial risk of primary hazards increase when the job step is performed in a particular weather condition?
	CQ 9	Will the initial risk of primary hazards increase when the job step is performed in a particular proximity condition?
	CQ 10	Will the initial risk of primary hazards increase when the job step is being performed in a particular atmospheric condition?

**Table 3:** Interviewee profile.

Interviewee ID	Designation	Experience (Years)	Company	Involvement in the JHA Process	
				Direct	Indirect
1	Supervisor	28	A	✓	
2	Supervisor	24	A	✓	
3	Supervisor	32	A	✓	
4	Safety consultant	27	A		✓
5	Supervisor	15	A	✓	
6	Supervisor	5	A	✓	
7	Supervisor	18	A	✓	
8	Site safety advisor	12	B	✓	
9	Senior safety advisor	21	B		✓
10	Safety manager	27	B		✓
11	Health and safety consultant	14	B		✓
12	HSEQ advisor	7	C	✓	
13	Safety manager	23	C		✓
14	HSEQ administrator	12	D		✓
15	HSEQ manager	34	D		✓
16	Supervisor	14	D	✓	
17	Safety consultant	30	E		✓
18	Field operation implementation manager	22	E	✓	

**Table 4:** Example of rules for risk evaluation.

Rule Category	Explanation	Graql Code Examples for Implementing Rules
Workplace-primary hazards risk rule	When a job step is performed in a particular workplace, such as a confined space and elevated work platform, it can increase the probability of primary hazards. This rule type evaluates the primary hazard and the workplace together during a query time and indicates that there is an increased risk of primary hazard due to the existing workplace condition	Example—High_risk_wp_9: when {$ph isa primary_hazards, has outcome_ph “breathing difficulties”, has consq_ph “high”; $wp isa workplace, has name_wpc “confined space”;} then {(high_risk_creator: $wp, high_risk_hazard: $ph) isa high_risk_relationship_wp;};
Weather-primary hazards risk rule	Existing weather conditions can largely increase the probability of the occurrence of a primary hazard. Even though it is not applicable to each hazard, the risk of some hazards can largely increase with the existing weather condition. This rule will go through the primary hazards of the job step and the existing weather condition and indicates the hazards which are likely to increase their risks.	Example—High_risk_we_5: when {$ph isa primary_hazards, has outcome_ph “electrocution”, has consq_ph “high”; $we isa weather, has name_wec “rainy”;} then {(high_risk_creator: $we, high_risk_hazard: $ph) isa high_risk_relationship_we;};
Atmosphere-primary hazards risk rule	Irrespective of creating its own set of hazards, atmosphere can influence the risk of primary hazards by increasing the probability of the occurrence of it. This rule will activate at the moment of querying and indicates to the user the primary hazards that can have an increased risk with the prevailing atmospheric condition	Example—High_risk_atm_2: when {$ph isa primary_hazards, has outcome_ph “cuts”, has consq_ph “high”; $atm isa atmosphere, has name_atm “dark”;} then {(high_risk_creator: $atm, high_risk_hazard: $ph) isa high_risk_relationship_atm;};
Proximity-primary hazards risk rule	The proximity condition can have some considerable influence on the risk of primary hazards by increasing the probability of occurrence. This rule will evaluate the primary hazards and the condition of the proximity at which the job step is being undertaken and highlights the hazards that are likely to have an increased risk as a result of the existing proximity.	Example—High_risk_pro_3: when {$ph isa primary_hazards, has outcome_ph “ground collapse”, has consq_ph “high”; $pro isa proximity, has name_pro “near a shaft or trench”;} then {(high_risk_creator: $pro, high_risk_hazard: $ph) isa high_risk_relationship_pro;}

**Table 5:** The result of Query 1, in a tabular form.

**Hazard**	Spark	Heat	Noise	Electricity	Asphyxiation Hazard
**Outcome**	Burns	Heat stress	Hearing loss	Electrocution	Breathing difficulties
**Consequence**	High	High	Medium	Medium	Medium

**Table 6:** The result of Query 2, in a tabular form.

**Hazard**	Ground Collapse	Underground Water
**Consequence**	High	Medium

**Table 7:** The result of Query 3, in a tabular form.

Control Measures	Category
All stockpiles should be inspected daily and particularly after heavy rain and earthquakes	Administrative control measures
Use stable and firm ground for stockpiling	Administrative control measures
Make sure the size of the area is sufficient for mobile equipment operation	Administrative control measures
Construct the ramp using a front-end loader, bulldozer, or other suitable machine and compact adequately	Engineering control measures
Maintain the ramp of the stockpile in a safe angle	Engineering control measures
Construct bunds at the edges of stockpiles	Engineering control measures

**Table 8:** The result of Query 4, in a tabular form.

Hazard Category	Hazard	Control Measures	Control Measures Category
High Risk Primary hazard	Heat	Take Regular Breaks	Administrative control measures
Workplace hazard	Musculoskeletal disorders	Reduce working time	Administrative control measures

## Data Availability

Not applicable.
